# Intra-articular injection of mesenchymal stem cells leads to reduced inflammation and cartilage damage in murine antigen-induced arthritis

**DOI:** 10.1186/1479-5876-12-157

**Published:** 2014-06-03

**Authors:** Oksana Kehoe, Alison Cartwright, Ayman Askari, Alicia J El Haj, Jim Middleton

**Affiliations:** 1 Keele University, ISTM at RJAH Orthopaedic Hospital, Oswestry SY10 7AG, Shropshire, UK; 2 RJAH Orthopaedic and District Hospital, Oswestry, Shropshire, UK; 3 Keele University, ISTM, Hartshill, Stoke on Trent, Staffordshire, UK; 4 University of Bristol, Faculty of Medicine and Dentistry, School of Oral and Dental Sciences, Bristol, UK; 5 Current address: Oxford University, Kennedy Institute of Rheumatology, London, UK

**Keywords:** Mesenchymal stem cells, Antigen-induced arthritis, Intra-articular administration, Chondroprotective, Anti-inflammatory, Tumour necrosis factor (TNF)α

## Abstract

**Background:**

Rheumatoid arthritis (RA) is a debilitating and painful disease leading to increased morbidity and mortality and novel therapeutic approaches are needed. The purpose of this study was to elucidate if mesenchymal stem cells (MSCs) injected in the joints of mice with arthritis are therapeutic, reducing joint swelling and cartilage destruction.

**Methods:**

Murine mesenchymal stem cells (mMSCs) were isolated from bone marrow of C57Bl/6 mice and expanded in culture. Cells were tested for immunophenotype and their ability to form colonies and to differentiate into chondrocytes, osteocytes and adipocytes. Antigen-induced arthritis (AIA) was induced by intra-articular injection of methylated bovine serum albumin into the knee joints of preimmunized C57Bl/6 mice. After one day, when peak swelling occurs, 500,000 mMSCs labelled with red fluorescent cell tracker CM-DiI were injected intra-articularly in the right knee joint. Left knee joints were treated as controls by receiving PBS injections. Differences between groups were calculated by Mann Whitney U test or unpaired t tests using GraphPad Prism software version 5.

**Results:**

Knee joint diameter (swelling) was measured as a clinical indication of joint inflammation and this parameter was significantly less in MSC-treated mice compared to control-treated animals 48 hours after arthritis induction. This difference continued for ~7 days. CM-DiI-labelled MSCs were clearly visualised in the lining and sublining layers of synovium, in the region of the patella and femoral and tibial surfaces. By day 3, parameters indicative of disease severity, including cartilage depletion, inflammatory exudate and arthritic index were shown to be significantly reduced in MSC-treated animals. This difference continued for 7 days and was further confirmed by histological analysis. The serum concentration of tumour necrosis factor α was significantly decreased following MSC administration.

**Conclusions:**

Our results reveal that MSCs injected in the joints of mice with AIA are therapeutic, reducing inflammation, joint swelling and cartilage destruction. These cells also integrate into the synovium in AIA.

## Background

Rheumatoid arthritis (RA) is a debilitating and painful disease leading to increased morbidity and mortality and novel therapeutic approaches are needed. Recent advances using biologic drugs, such as anti-TNF, have made a significant impact on the treatment of RA patients although many patients do not respond and 50% discontinue the drug after 2 years [1]. For that reason, it is vital to develop a new and more effective therapy for RA.

Mesenchymal stem cells (MSCs) show therapeutic potential in pre-clinical models of inflammatory diseases and in some clinical trials in patients with Crohn’s disease, diabetes, GVHD and myocardial infarction [1]. Recently, the first human trial using umbilical cord mesenchymal stem cells (UC-MSCs) was published for RA and the results confirmed the safety and efficacy of UC-MSC infusion in active RA patients [2]. MSCs are an ideal candidate cell type for tissue engineering and cell therapy to repair damaged structures in various arthritic conditions. MSCs possess anti-inflammatory and immunosuppressive properties modulated by the secretion of biologically active molecules [3] and since RA is a chronic inflammatory autoimmune disease involving tissue destruction, the anti-inflammatory and regenerative functions of MSCs could be exploited as a therapy [4]. MSCs have been given intravenously or intraperitonealy in animal models of RA and lead to different therapeutic effects, varying from significant improvement to no effect so overall the results remain inconclusive [5]. The reason for this may be the route of administration. A number of studies used intravenous or intraperitoneal administration and the MSCs were not reported to migrate into the joints to exert their effects, but have been located in the spleen [3,4]. Intra-articular administration of MSCs may be more beneficial than the intravenous/intraperitoneal route, applying them directly to the affected tissues.

Here, we investigated whether intra-articular injected MSCs are therapeutic, reducing joint swelling and cartilage destruction in murine antigen-induced arthritis (AIA). The characteristics of this model are as follows [6-9]: preimmunisation induces humoral and cell-mediated immunity; leukocyte migration occurs into the joint, including neutrophils, lymphocytes, plasma cells and macrophages; it is a uniarthritis with controlled onset; pannus formation occurs resulting in erosive changes of cartilage and bone; it is antigen-specific with local hyper-reactivity and antigen retention in cartilage; chronicity can be achieved by repeated flares.

## Methods

### Animals

Experiments were undertaken in 7- to 8-wk-old C57Bl/6 male mice. Procedures were performed in accordance with Home Office-approved project licence PPL 40/3594.

### Induction of murine AIA

Murine AIA was induced as described [6]. Briefly, mice were immunised subcutaneously with 1 mg/ml of methylated BSA (mBSA) emulsified with an equal volume of Freund’s complete adjuvant and injected intraperitonealy with 100 μl heat-inactivated *Bordetella pertussis* toxin (all reagents were from Sigma-Aldrich). The immune response was boosted 1 week later. Twenty-one days after the initial immunisation, murine AIA was induced by intra-articular injection of 10 mg/ml mBSA in the right knee (stifle) joint. For a control, the same volume of PBS was injected into the left knee joint. Animals were inspected daily for arthritis development by measuring knee joint diameters using a digital micrometer (Kroeplin GmbH). The difference in joint diameter between the arthritic (right) and non-arthritic control (left) in each animal gave a quantitative measure of swelling (in mm).

### Cells

Murine MSCs (mMSCs) were isolated from C57Bl/6 mice (n = 5) as previously described [10]. Briefly, bone marrow cells were collected by flushing them out of femurs and tibiae and cells plated out in cell isolation media (CIM) (RPMI-1640) (Gibco,UK) with 9% fetal bovine serum (Gibco,UK), 9% horse serum (Gibco, UK) and 1% penicillin-streptomycin at 37°C in 5% CO_2_. After 24 hours, nonadherent cells were removed and 4 weeks later, cells were re-plated at 100 cells per cm^2^ in complete expansion media (CEM) (Iscove Modified Dulbecco Medium (IMDM)) (Gibco, UK) supplemented with 9% fetal bovine serum (Gibco, UK), 9% horse serum (Gibco, UK) and 1% penicillin-streptomycin for MSC expansion. Cells were examined for their ability to differentiate into chondrocytes (using pellet cultures), osteocytes and adipocytes, as described [10].

For the colony-forming unit assay 100 cells at passage 3 were plated in triplicate on 58 cm^2^ plates in CEM as described in [10]. Cells were incubated for 14 days in CEM, and stained with 3% crystal violet in methanol at room temperature for 20 minutes. All visible colonies were counted. Passage 3 cells were tested for immunophenotype with the following antibodies: anti-mouse Ly-6A (Sca-1)PE, anti-human/mouse CD44 PE, anti-mouse CD11b PE, anti-mouse CD45 PE and anti-mouse CD31 PE (all from eBioscience) using flow cytometry. Data were collected and displayed in dot plot and histogram format using CellQuestPro software (Becton Dickinson, Oxford, UK). More than 95% of mMSCs were positive for Sca-1 (murine MSC marker) and CD44 (mesenchymal cell marker) cell surface markers and were less than 3% positive for CD11b (macrophage and monocyte marker), CD45 (leukocyte marker) and CD31 (endothelial cell marker). Propidium iodide staining was included in the immunophenotyping to evaluate the viability of the cells.

### CM-DiI labelling

A stock solution of the red fluorescent cell tracker CM-DiI (Molecular Probes, UK) was prepared in dimethyl sulfoxide (DMSO) at concentration of 1 mg/ml. MSCs were trypsinized, washed with phosphate-buffered saline (PBS), and incubated in the working solution of CM-DiI (2.5 μl of stock per 1 ml of PBS) for 5 minutes at 37°C, and then for additional 15 minutes at 4°C, in the dark. Unincorporated dye was then removed by centrifugation at 300 g for 5 minutes and 2 washes in PBS. Cells were resuspended in serum free IMDM and maintained at 4° until injection.

### Intra-articular injection of MSCs

After one day post arthritis induction, when peak swelling occurs, 10 μl of serum free IMDM, containing 500,000 MSCs labelled with cell tracker CM-DiI were injected intra-articularly (0.5 ml monoject (29 G) insulin syringe, BD Micro-Fine, Franklyn Lakes, USA) through the patellar ligament into the right knee joint. Stretching of the hind-leg facilitated the intra-articular injection. Control animals were injected with 10 μl of serum free IMDM. Joint diameters were measured at days 1, 2, 3, 5, 7, 14, 21 and 28. At the end of the experiments, animals were killed and joints were collected for histology. Experiments were performed independently, twice, and all measures were made to reduce the number of used animals (n = 6 animals/per time point).

### Histological assessment

Animals were sacrificed at the indicated times after induction of arthritis (at days 3, 7, 14, 21 and 28; n = 6 animals/per time point). Joints were fixed in neutral buffered formal saline, and decalcified with formic acid at 4°C before embedding in paraffin. Mid-sagittal serial sections (5 μm thickness) were cut and stained with haematoxylin and eosin (H&E). For detection of CM-DiI-labelled MSCs, sections were rehydrated through a xylene and alcohol, stained with fluorescent dye DAPI (Sigma-Aldrich, UK), mounted in Hydromount (National Diagnostics, UK) and examined by fluorescence microscopy. Two independent observers blinded to the experimental groups scored H&E sections. Synovial hyperplasia, cellular exudate and cartilage depletion were scored from 0 (normal) to 3 (severe); synovial infiltrate was scored from 0 to 5 [6]. Cartilage damage was scored on serial toluidine blue stained sections. All parameters were subsequently summed to give an arthritis index (mean ± SEM).

Endothelial cells in the joint synovia were identified by immunofluorescence with rabbit anti-von Willebrand factor (1:100; Dako, Ely, UK) followed by goat anti-rabbit Alexa 488 second antibody (1:400; Life Technologies, Paisley, UK).

### TNFα assay

Serum concentration of TNFα was measured using a mouse TNFα ELISA Ready-SET-Go! Kit (eBioscience) according to the manufacturer’s instructions.

### Statistical analysis

Differences between groups were compared by Mann Whitney U or unpaired t tests, using GraphPad Prism software version 5*. P* values less than 0.05 being deemed as significant.

## Results

### Characterisation of murine bone marrow MSCs

**Figure 1:**
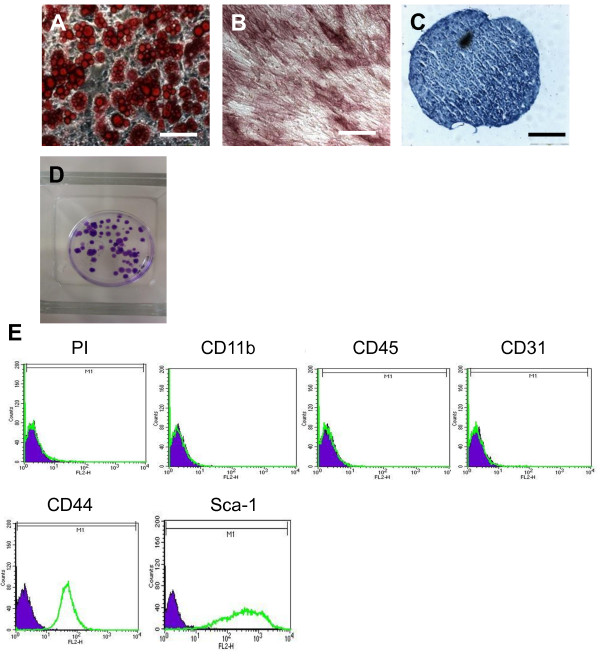
**In vitro characterisation of murine MSCs (mMSCs).** Passage 3 mMSCs underwent successful differentiation down adipogenic **(A)**, osteogenic **(B)** and chrondrogenic **(C)** lineages after 21 days in appropriate differentiation media. **D)** Representative colony-forming unit analysis. **E)** Immunophenotypic profile of passage 3 mMSCs are negative for haematopoietic markers CD11b and CD45, endothelial marker CD31 and positive for mesenchymal markers CD44 and Sca-1. The solid peak is corresponding isotype control. Scale bars = 100 μm in A and B, 200 μm in C.

mMSCs were successfully differentiated towards adipogenic, osteogenic and chondrogenic lineages after 21 days in culture with relevant differentiation media (Figure 1A-C). Adipogenic differentiation was verified by the presence of lipid droplets stained positive with oil red O (Figure 1A), the osteogenic differentiation was detected by alkaline phosphatase (ALP) staining (Figure 1B) and chondrogenesis was confirmed by formation of dark blue/purple proteoglycan-producing pellets (Figure 1C). Colony-forming units assay was used to assess the proliferative capacity of the cells being expanded in culture, and the results showed that passage 3 cells retained the high proliferation rate in culture (Figure 1D).Flow cytometry analysis showed that isolated and cultured mMSCs were negative for hematopoietic markers CD11b and CD45, the endothelial cell marker CD31 (PECAM), and positive for the mesenchymal markers CD44 and Sca-1 (Figure 1E). Propidium iodide staining indicated a cell viability >96%.

### Decreased severity of AIA following treatment with murine MSCs

**Figure 2:**
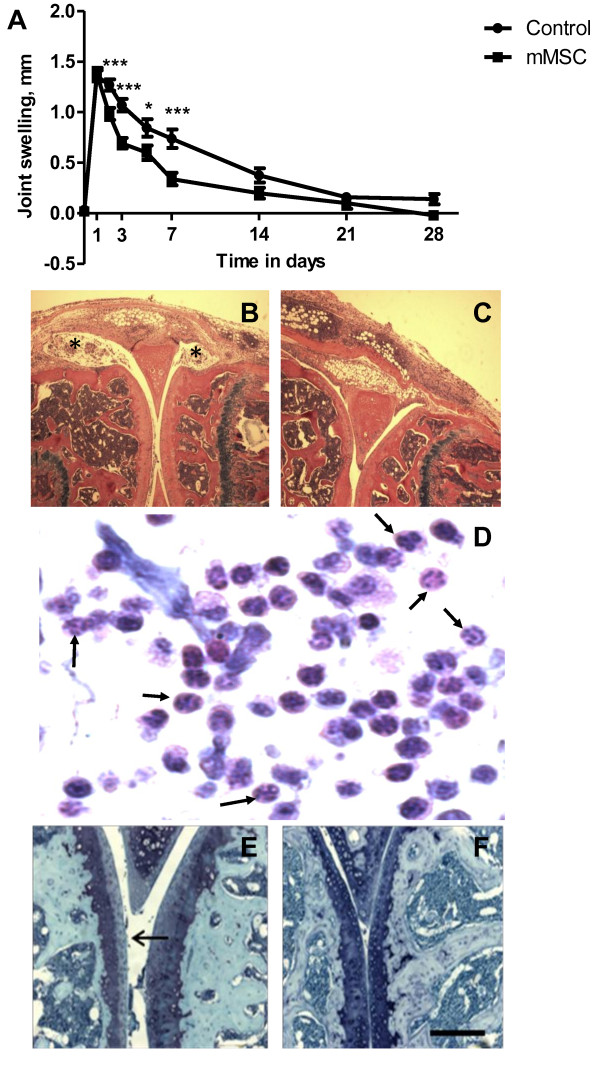
**The effects of intra-articular injection of murine MSCs (mMSCs) into knee joints of mice with antigen-induced arthritis.** Arthritis was induced by intra-articular injection of methylated BSA in the right knee (stifle) joint (t = 0 in A). For a control, PBS was injected into the left knee joint. Murine MSCs were isolated from bone marrow and expanded in culture. 500,000 mMSCs in serum free medium were injected at the peak of joint swelling (after 1 day) into the knee joints of mice with arthritis. Injection of serum free medium alone was control. **(A)** Reduced joint swelling in the presence of mMSCs. Joint diameters were measured as an index of swelling (joint inflammation). Data are means ± SEM for right knee after subtraction of left knee control; n = 6 mice with MSCs and control without MSCs. *p < 0.05, ***p < 0.001 compared with corresponding time point. **(B-F)** Histology of joints in mouse antigen-induced arthritis 3 days after induction. **(B)** Arthritic knee joint with marked leukocyte exudates in the joint space (*). **(C)** is the same as B except MSCs have been injected. Note the lack of exudates. **(D)** detail of exudate in the joint cavity in the absence of MSCs comprising leukocytes, particularly neutrophils (arrows show examples). **(E)** arthritic knee joint with loss of proteoglycan staining in the surface regions of tibial and femoral articular cartilage (arrow). F is the same as E except the joint was injected with mMSCs and there is less cartilage degradation. B, C and D were stained with haematoxylin and eosin and E and F with toluidine blue. Scale bar =500 μm in B and C, 20 μm in D and 200 μm in E and F.

AIA is model of inflammatory arthritis with many histopathological and clinical similarities to RA [6-9]. In order to investigate the therapeutic effects of mMSCs we used AIA which was induced in the right knee joints of C57Bl/6 mice (t = 0). After one day, when peak swelling occurs, the knee joints were injected with mMSCs. Knee joint diameters (swelling) were measured as a clinical indication of joint inflammation and this parameter (mean ± SEM in mm) was significantly less in MSC-treated mice compared to control-treated animals 2 days after arthritis induction (0.98 ± 0.06 mm versus 1.27 ± 0.06 mm; p = 0.0009) (Figure 2A). This difference continued for ~7 days post intra-articular mBSA administration (0.34 ± 0.06 mm versus 0.74 ± 0.09mm; p = 0.0009). At days 14 and 21 there were no significant differences in swelling between MSC-treated and non-treated animals.

**Figure 3:**
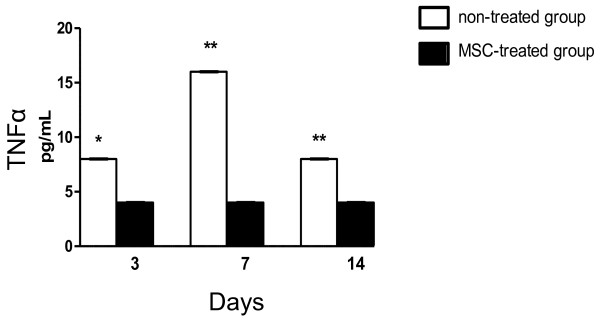
**Serum concentration of TNFα at the indicated time points (day 3**, **7 and 14)**, **analysed by enzyme-linked immunosorbent assay.** Data are means ± SEM; n = 6 mice with MSCs and control without MSCs. *p < 0.05, **p < 0.01.

Histologically AIA was characterised by synovial hyperplasia of the synovial lining layer, infiltration of the synovial sublining by leukocytes, exudate in the joint cavity, and loss of proteoglycans from the articular cartilage, as observed in haematoxylin/eosin and toluidine blue stained sections (Figure 2B, D and E). These changes did not occur in contralateral knee joints which were injected with PBS instead of mBSA and appeared histologically normal. The inflammation and cartilage degradation appeared less severe in MSC-treated mice compared to control treated (Figure 2B-F). In order to quantitate these changes, the parameters were scored as a measure of disease severity and differences between MSC-treated and non-treated mice were evident (Table 1). In MSC-treated mice at day 3 there was a significant reduction of amount of exudates in the joint cavity which contained leukocytes including neutrophils (p = 0.0004), cartilage depletion (p = 0.0003) and arthritis index representing overall disease severity (p = 0.0009) (Table 1, Figure 2B-F). At day 7 post intra-articular injection of mBSA, there were still significant differences between MSC-treated and non-treated mice for exudate (p = 0.024), cartilage depletion (p = 0.035), synovial hyperplasia (p = 0.01) and arthritis index (p = 0.013). At day 14, all parameters were reduced in the presence of MSCs although these only approached significance (eg p = 0.09 for cartilage depletion) (Table 1). At day 28 post intra-articular injection of mBSA, there were no significant differences between MSC-treated and control-treated animals for all parameters.Serum levels of TNFα were lower in the MSC-treated group with AIA compared to the non-treated group at day 3 (p = 0.024), day 7 (p = 0.001) and day 14 (p = 0.008) (Figure 3).

**Table 1:** **Joint inflammation and cartilage damage on day 3**, **7 and 14 of antigen-induced arthritis as assessed by histological scoring**

**Species**	**Days**	**Hyperplasia**	**Synovial infiltrate**	**Exudate**	**Cartilage depletion**	**Arthritis index**
Control animals	3	1.75 ± 0.29	3.56 ± 0.40	2.78 ± 0.15	1.78 ± 0.33	9.89 ± 0.88
	7	2.43 ± 0.23	3.93 ± 0.39	2.14 ± 0.46	1.71 ± 0.52	10.2 ± 1.46
	14	1.67 ± 0.67	1.67 ± 1.20	1.17 ± 0.92	1.50 ± 0.87	6.00 ± 3.61
MSC treated animals	3	1.70 ± 0.17	2.83 ± 0.25	1.13 ± 0.32***	0.33 ± 0.14***	5.96 ± 0.57***
	7	1.19 ± 0.28*	2.69 ± 0.43	0.75 ± 0.31*	0.44 ± 0.29*	5.06 ± 1.09*
	14	1.00 ± 0.00 (p = 0.39)	1.00 ± 0.41 (p = 0.58)	0.00 ± 0.00 (p = 0.19)	0.00 ± 0.00 (p = 0.09)	2.00 ± 0.41 (p = 0.25)

Synovial hyperplasia of the lining layer, synovial infiltration of the sublining by leukocytes, exudate in the joint cavity, and loss of proteoglycan from the articular cartilage were observed in haematoxylin/eosin and toluidine blue stained sections. Sections were scored blind by two independent observers from 0 to 3 (hyperplasia, exudate and cartilage depletion) or 0 to 5 (infiltrate). The arthritis index is the sum of all observations. *p < 0.05; and ***p < 0.001 of the parameter compared to control animals at the same point, using unpaired t test. n = 6 mice.

### Intra-articularly injected MSCs migrated into the synovia of knee joints

**Figure 4:**
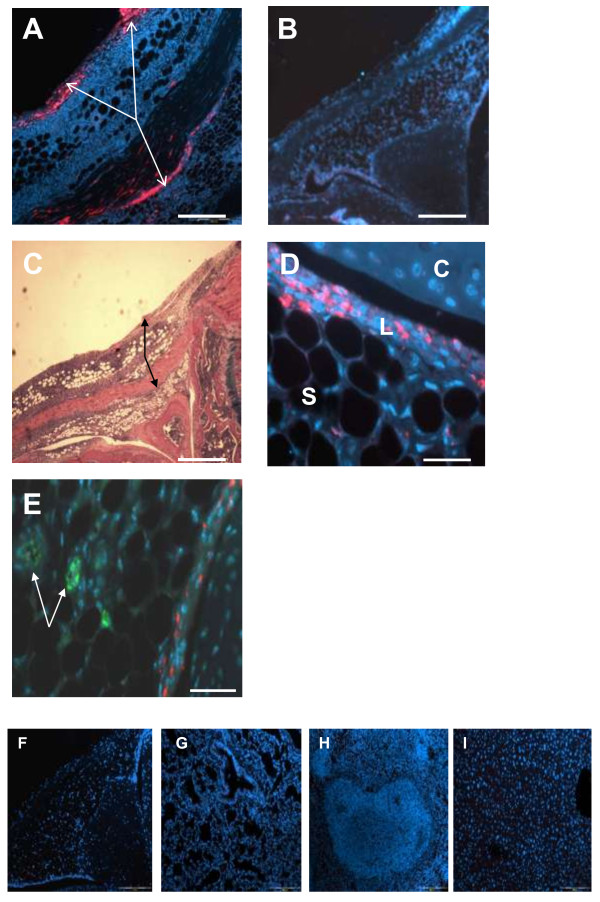
**MSCs injected into the joint localise to the synovium.** 500,000 CM-DiI-labelled red-fluorescent mouse MSC were injected intra-articularly into the knee joint spaces of mice with antigen-induced arthritis. **(A)** low magnification image of a mouse joint 2 days after MSC injection, the arrows indicate MSCs (red) localising to the synovium. **(B)** joint of control treated mouse 2 days after MSC injection, no red fluorescence, DAPI blue. **(C)** low magnification H&E stained image of a mouse joint 2 days after MSC injection (same joint as A), the arrows indicate regions of MSC localisation (corresponding to A). **(D)** higher magnification showing MSCs localising to the lining of synovium (C-cartilage; L-lining layer of the synovium; S-sublining layer of the synovium). DAPI stain cell nuclei. **(E)** sections were treated with rabbit anti- von-Willebrand factor antibodies followed by goat anti-rabbit Alexa 488 to identify endothelial cells (arrows) in MSC-injected joints. Absence of red fluorescence in **F**- contra-later joint, **G**- lungs, **H**-spleen and **I**-liver. Scale bar 500 μm in **A, B, C;** 50 μm in **D** and **E;** and 200 μm in **F-I**.

3, 7, 14 and 28 days after arthritis induction CM-DiI-labelled MSCs were clearly visualised in the lining and sublining layers of the synovium, in the region of the patella, and femoral and tibial surfaces (Figure 4A and D). Figure 4E illustrates absence of MSC-endothelial cell co-localisation in the synovium of injected joints. At all time points there was no localisation of labelled MSCs in other joint tissues such as articular and meniscal cartilage. To further assess whether injected MSCs could migrate to some distant organs or not, we tried to detect CM-DiI-labelled red-fluorescent MSCs by immunofluorescence in contralateral joints and in lungs, spleens and livers of mice at the end of the experiments. We were unable to identify any red-fluorescent cells in these organs at different time points during the experiment (Figure 4F-I).

## Discussion

In this study, we examined a therapeutic strategy for arthritis using a single intra-articular injection of MSCs. We found that these cells are therapeutic, reducing the severity of AIA in mice. An injection of 500,000 MSC given at the peak of joint swelling was enough to prevent the occurrence of severe cartilage damage, and reduce joint inflammation and exudate in the joint cavity. Studies of MSCs as a cellular therapy for animal models of RA exhibit contradictory results [5]. Experimental protocols differed between all of these studies, which may, in part, explain discrepancies in results.Djouad et al, Mao et al and Choi et al used intravenous but González et al. used intraperitoneal administration and the MSCs were not reported to migrate into the joints to exert their effects, but have been located in the spleen [5,11-13]. Furthermore intravenously injected MSCs are known to become lodged in the lungs which could further hamper their therapeutic effect [14]. Our data suggest that intra-articular administration of MSCs may be more beneficial than the intravenous/intraperitoneal route, applying them directly to the affected tissues. The homing of systemically injected MSCs in the AIA model has not been studied and will be of the interest to compare with the results of intra-articular administration used in the present study and to compare with other models such as systemic injections in collagen-induced arthritis. Our findings indicate that joint swelling in arthritis as a clinical indication of joint inflammation is reduced in the presence of administered MSCs and these cells migrate into the inflamed synovium. Furthermore, MSCs reduce the amount of exudates in the joint cavity. Joint swelling is common with different types of arthritis and is caused by oedema due to the endothelial cells of blood vessels becoming leaky in the inflamed synovium [15]. We hypothesise that soluble factors produced by MSCs are responsible for permeability changes in the synovial endothelial cells, although further studies are required in this regard. Direct MSC-endothelial cell contact is most likely not responsible since these cells did not colocalise (Figure 4E). In this connection, in vitro and in vivo studies with pulmonary endothelial cells reveal that MSCs and conditioned media from these cells inhibit endothelial cell permeability and lung oedema by preserving adherent junctions (VE-cadherin and β-catenin) [16]. Recent studies show that complexes of alarmins S100A8 and S100A9, which are major products of neutrophils and macrophages [17], bind to endothelial cells via specific interaction with heparan sulphate proteoglycans, inducing inflammatory responses in endothelial cells and increasing the endothelial permeability [18]. It is currently unknown if mMSCs and alarmins effect permeability of the synovial endothelial cells.

Histological analysis of joint sections was used to determine the nature of cells in exudate within the joint space. Leukocytes, and in particular neutrophils, were identified by their nuclear morphology and were broadly distributed throughout joint exudate in control-treated mice. There was a significant reduction of amount of exudates, comprising leukocytes in the joint cavity in MSC-treated mice at days 3 and 7 after arthritis induction. Neutrophils have been considered as important cells in the development of inflammatory joint disease, as supported by several studies involving experimental models of arthritis [19]. Neutrophils are found in high numbers within the human rheumatoid joint, especially in the joint fluid. Here they have a significant potential to directly inflict damage to tissue, bone and cartilage via the secretion of proteases and toxic oxygen metabolites, as well as driving inflammation through antigen presentation and secretion of cytokines, chemokines, prostaglandins and leucotrienes [20]. Neutrophil depleted mice are completely resistant to the inflammatory effects of arthritogenic serum from K/BxN mice [19]. In the present study an intra-articular injection of MSCs given at the peak of joint swelling reduces the accumulation of leukocytes in the joint fluid in AIA which may be related to the reduced joint damage in terms of cartilage depletion. It is possible that soluble anti-inflammatory factors produced by MSCs influence leukocyte accumulation in the joint fluid during inflammation in AIA. In the synovium there was also a reduction in leukocyte infiltration but this did not reach statistical significance.

Cartilage damage was scored on serial toluidine blue–stained sections based upon proteoglycan loss in articular cartilage. Healthy cartilage shows a dark blue stain and a loss of cartilage proteoglycans is indicated by cartilage destaining [21].

We directly compared the extent of cartilage damage in control and MSC-injected sections by semi-quantitative histological scoring (Table 1) and cartilage depletion was scored from 0 (normal) to 3 (severe).

Our results showed inhibition of proteoglycan loss, a marker of early cartilage destruction, by local MSC treatment. Breakdown of the cartilage matrix is one of the features of RA. Aggrecan can be cleaved by both matrix metalloproteinases (MMPs) and a disintegrin and metalloproteinase with a thrombospondin type 1 motif (ADAMTS) at different sites. Formation of the NITEGE (ADAMTS-cleaved) and DIPEN (MMP-cleaved) aggrecan neoepitopes and their detection with neoepitope antibodies can provide information about which enzyme is the major one that degrades aggrecan in mouse cartilage and has set a target for the development of new drugs designed to inhibit cartilage destruction in RA [22].

Early cartilage destruction in AIA is characterised by loss of aggrecan and the ADAMTS family members are thought to be involved in mediating this loss [18,23]. ADAMTS-5 is of special interest since only lack of the *Adamts-5* gene prevents cartilage damage in a mouse model of arthritis [24]. It is possible that factors released by MSCs influence the activity or expression of ADAMTS enzymes resulting in less aggrecan degradation. It has been shown in experimental osteoarthritis model that stem cell treatment strongly inhibited expression of neo-epitopes (NITEGE), suggesting suppression of ADAMTS activity [17]. Another possibility is that MSCs are directly differentiating into chondrocytes leading to less cartilage destruction. However, this is unlikely as injection of fluorescently labelled MSCs did not localise to cartilage but to the inflamed synovium (Figure 4A and 4D).

TNFα is a cytokine involved in inflammation and tissue degradation in RA. The finding that administration of MSCs results in reduced levels of TNFα in the circulation may be related to their anti-inflammatory effects, in terms of reducing oedema, swelling and leukocyte accumulation, and cartilage protective effects namely inhibiting proteoglycan degradation.

Several groups demonstrated therapeutic effects of intra-articular injections of MSCs in experimental osteoarthritis models [17,25-27]. These studies showed anti-inflammatory and reparative effects on cartilage using both bone marrow-derived and adipose-derived stem cells. In addition, intra-articularly injected adipose-derived stem cells were found to home to the subintimal synovial lining layer in mice [17]. The current study in an RA model is in general agreement with these osteoarthritis models.

## Conclusion

Our data demonstrate that an intra-articular injection of MSCs into the knee joints of mice with AIA ameliorates the severity of disease, causing reduced swelling, exudates in the joint fluid and cartilage damage. These MSCs home to the synovium.

## Competing interests

The authors declare that they have no competing interests.

## Authors’ contributions

OK performed the experimental work and wrote the paper; AC performed the experimental work; AA, AEH and JM designed the study and JM also wrote the manuscript. All authors read and approved the final manuscript.
